# Modeling the role of police corruption in the reduction of organized crime: Mexico as a case study

**DOI:** 10.1038/s41598-022-23630-x

**Published:** 2022-11-10

**Authors:** Andrés Aldana, Hernán Larralde, Maximino Aldana

**Affiliations:** 1 Instituto de Biotecnología, Universidad Nacional Autónoma de México, Cuernavaca, MOR Mexico; 2 Centro de Ciencias de la Complejidad, Universidad Nacional Autónoma de México, Ciudad de México, Mexico; 3 Instituto de Ciencias Físicas, Universidad Nacional Autónoma de México, Cuernavaca, MOR Mexico

**Keywords:** Complex networks, Nonlinear phenomena, Statistical physics

## Abstract

Among all types of corruption, police corruption is probably the one that most directly hurts society, as those trusted with protecting the people either side with the criminals that victimize the citizens, or are themselves, criminals. However, both corruption and its effects are very difficult to measure quantitatively other than by perception surveys, but the perception that citizens have of this phenomenon may be different from reality. Using a simple agent-based model, we analyze the effect on crime rates as a result of both corruption and the perception of corruption within law-enforcement corporations. Our results show a phase transition in which crime can propagate across the population even when the majority of police officers are honest. We find that one of the parameters that strongly controls crime incidence is the probability that regular citizens become criminals. In contrast, other actions, such as arresting crime lords, or the amount of crime-associated money that is confiscated, have little impact on the long-term crime incidence. Our results suggest that in addition to combating corruption within law-enforcement institutions, to further reduce the incidence of crime, policymakers should strive to restore confidence in these institutions and the justice system.

## Introduction

Corruption is very difficult to measure, since not many corporations, agencies, or institutions, are willing to openly report acts of corruption within their ranks. Therefore, it has to be estimated by indirect means. One of the most prominent institutions estimating corruption worldwide is Transparency International, which has developed the *Corruption Perception Index* (CPI) based on more than 20 different parameters. The name for this index was carefully chosen, as it is not measuring the actual levels of corruption, but rather, the *perception* that people have about the levels of corruption in their societies. Determination of the CPI is based on surveys carried out in many regions around the world. It is noteworthy that in many developing countries, police (and more generally, law enforcement) corporations are perceived by society as extremely corrupt. This is due partly to the fact that indeed corruption in law enforcement officers is frequently widespread in those countries, causing great damage to the structure of society^1–3^, but also, partly due to the fact that the news related to acts of corruption within these corporations are often magnified by media and social networks^4–6^. Thus, the perception that people may have about corruption in police corporations (and in any other institution) need not correspond to its actual levels. For instance, according to the Global Corruption Barometer of the Americas and the Caribbean 2019^7^, on average 45% of the people in 18 American countries think that *most or all* of the police officers in their countries are corrupt. However, in the same survey Transparency International reports that only 26% (from 12% in Chile to 62% in Venezuela) of the people that had contact with police officers that year were victims of police corruption (specifically bribes). A rate of 26% is undoubtedly a large percentage, but does not appear to justify the perception, held by 45% of the society, that “most or all” police officers are corrupt.

This biased perception has an important consequence: When society considers that most or all police officers are corrupt, police corporations lose legitimacy to enforce the law^8,9^. This is the case of Mexico, a country afflicted by violence, in which police reportedly took bribes in 52% of the cases in which they interacted with people^7^, and between 72 and 77% of the citizens consider that most or all the police corporations are corrupt^7,10,11^. Indeed, it has been thoroughly documented by the Mexican and international press that police corporations in Mexico, particularly the local and state police, do not have legitimacy to fight crime, even when criminals are caught in *flagrante delicto*^12–14^. On the other hand, because of this biased perception, those police officers who are not corrupt cannot protect citizens from crime because they do not have the legitimacy to do so, reinforcing the lack of security in the society. Importantly, this perception may extend beyond the police corporations to other branches of the government^2,8,12–15^, as has been accurately pointed out by Hope^16^:

*“Acts of corruption by people in or with power have long shaken public faith in government, but the loss of public faith is particularly acute when those acts involve the police. That is so because the public relies on the police to uphold the law, protect the community, and assist it in times of need. The police are also the most visible arm of government for most citizens and a yardstick by which they measure authority. When a police officer acts illegally, he or she dishonors both himself or herself and the law and the justice system he or she represents.”*

Another harmful consequence of an inflated perception of police corruption is that it may enhance corrupt behavior in police officers, as has been proposed and corroborated within the context of the Labeling Theory of Criminology. This theory states that *labeling someone a criminal makes that person more prone to commit criminal acts*^17,18^. From this point of view, what motivation would some police officers have to behave honestly and protect a society that disrespects (and *a priori* criminalizes) most or all police officers because of an excessive perception of corruption levels? To break this vicious cycle, it is important to assess the actual levels of corruption in police (and law enforcement) corporations not just through the citizens’ perception. In this work we analyze the following problem: how do crime levels change as a function of police corruption and its perception?

We present a computational agent-based model to answer the previous question. Our model is simplified to introduce as few parameters as possible. However, we believe it captures important aspects of the corruption/crime dynamics. The most important parameter to control the crime level turns out to be the probability that an ordinary citizen becomes a criminal, which depends on the actual fraction of corrupt police officers and the perception of corruption. We also use a money-based preferential attachment algorithm to construct criminal networks and show that catching the kingpins (the “hubs”) of the network and confiscating their money only fractures the network down into smaller cells but does not reduce crime levels in the long run, which is consistent with empirical observations^19–25^. However, the crime level can be drastically reduced by decreasing (even by a small amount) the probability that regular citizens become criminals, which, as mentioned above, in turn depends on the perception of corruption in the society. Reduction in the crime level occurs through a phase transition driven independently by the fraction of corrupt officers and the probability for a regular citizen to become a criminal. This phase transition appears to be continuous, i.e. of second order. In the next section, we present the phenomenology on which our model is constructed. This phenomenology is based on data from Mexico, and shows that crime levels are considerably more (anti) correlated with citizens’ trust in the police and justice system, than with any other welfare indicator. Next, we present the model and the results. Finally, we discuss the importance of our findings for reconstructing police legitimacy and formulating strategies to reduce the level of crime.

## Model and results

### Phenomenological justification

**Figure 1:**
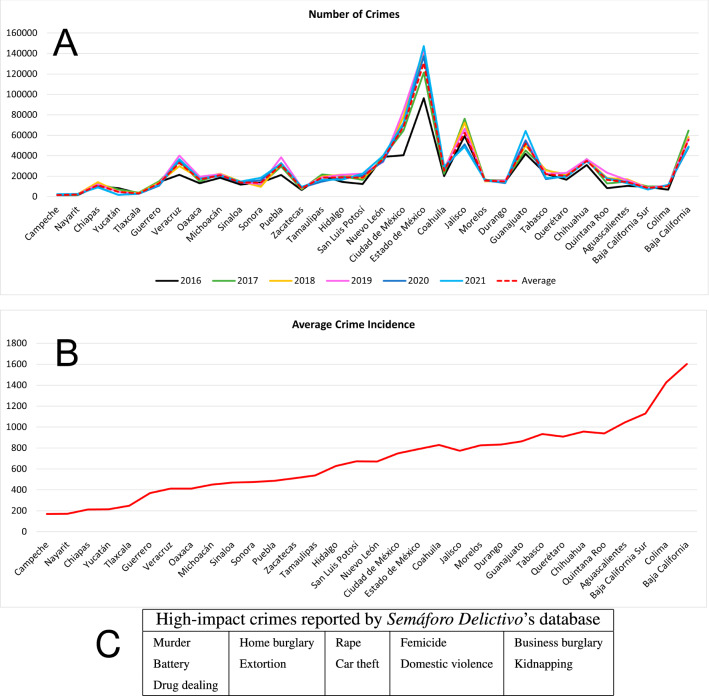
Crime levels in Mexico. (**A**) Number of crimes in each of the 32 Mexican States from 2016 to 2021. The red dotted line is the average over these six years. (**B**) Average crime incidence (number of victims per 100,000 inhabitants per year) for each Mexican State, shown in increasing tendency. The average is taken from 2016 to 2021. (**C**) High-impact crimes reported in the *Semáforo Delictivo* data base^26^ and used to construct the graphs in (**A**) and (**B**).

Figure 1A shows the number of high-impact crimes committed in each of the 32 Mexican States from 2016 to 2021, according to the data reported by *Semáforo Delictivo* (“Criminal Traffic Light”)^26^. This database, which we will refer to as SDDB, presents monthly data about the eleven high-impact crimes listed in the table shown in Fig. 1. There are two other main institutions registering crime in Mexico, each with its own database: the Department of Public Security (SSP)^27^ and the National Institute of Statistics and Geography (INEGI)^28^. Supplementary Fig. S1 shows that these three databases are consistent with each other. From Fig. 1A it can be observed that crime in Mexico has not significantly changed in the six years from 2016 to 2021. Figure 1B shows the average of the crime incidence over these six years for each State in Mexico. (Crime incidence is defined as the number of victims per 100,000 inhabitants per year.) It should be noted that the data presented in Fig. 1 do not include minor offenses such as shoplifting or bike theft, nor white-collar crimes. Although important, bike theft, shop lifting or white-collar corruption are not the crimes that regular citizens are afraid of in their everyday lives (at least not at the level of kidnapping or human trafficking, for instance). In what follows we will concentrate only on the crimes listed in Fig. 1, as such crimes are the ones that can be fought by operative policemen and make them prone to corruption (white-collar crime usually require special task forces).

To estimate the parameters of the model we will work with the average data of crime reported during the last three years, from 2019 to 2021. According to SDDB, the average number of high-impact crimes in Mexico in these three years was 844,281 (Supplementary Fig. S2A). The Mexican population is about 127.6 millions (which has not appreciably changed from 2019 to 2021). Therefore, the average percentage of victims in these three years was 0.66%. However, not every person in the society is a potential target for criminals. Rather, the *economically active people (EAP)* represent the most likely targets for criminals. This is supported by Supplementary Fig. S3, which shows that the majority of the crimes reported in Fig. 1 are money-related (except for domestic violence and femicide, being the former comparable in numbers to car theft whereas the later is marginal compared to all other types of crime). According to INEGI, the average number of EAP from 2019 to 2021 was 55,829,091.42 (Supplementary Fig. S2B)^29^. Therefore, the percentage of EAP who were victims of crime in each of these three years is close to 1.5%. This percentage could be ten times larger, as it has been estimated that in Mexico 90% of the crimes may not be reported (the so-called *dark figure of crime*; the two types of violent crime which are most accurately reported are murder and car theft; other types of crime, such as extortion, rape, kidnapping, domestic violence, burglary, street robbery or battery are the ones that contribute most to the dark figure. In Supplementary Fig. S1, car theft and murder were chosen to compare the three different databases mentioned in this work. This analysis shows great consistency among these databases.)^11^. Thus, the actual level of crime, considering only the EAP, is somewhere between 1.5 and 15% per year. We can consider these percentages as the lower and upper bounds, respectively, for the level of crime in Mexico.

One of the main problems faced by politicians, law makers, police authorities, and criminologists is to determine the causes of crime and corruption, particularly within police corporations. Crime and corruption are complex problems whose causes are multifactorial and depend on the context and region. Many hypothesis and models have been put forward such as low-income^30–32^, trust in the community^33^, low salaries of police officers^34^, etc. It has even been argued that corruption is a natural consequence of the evolution of cooperation^35,36^.

**Figure 2:**
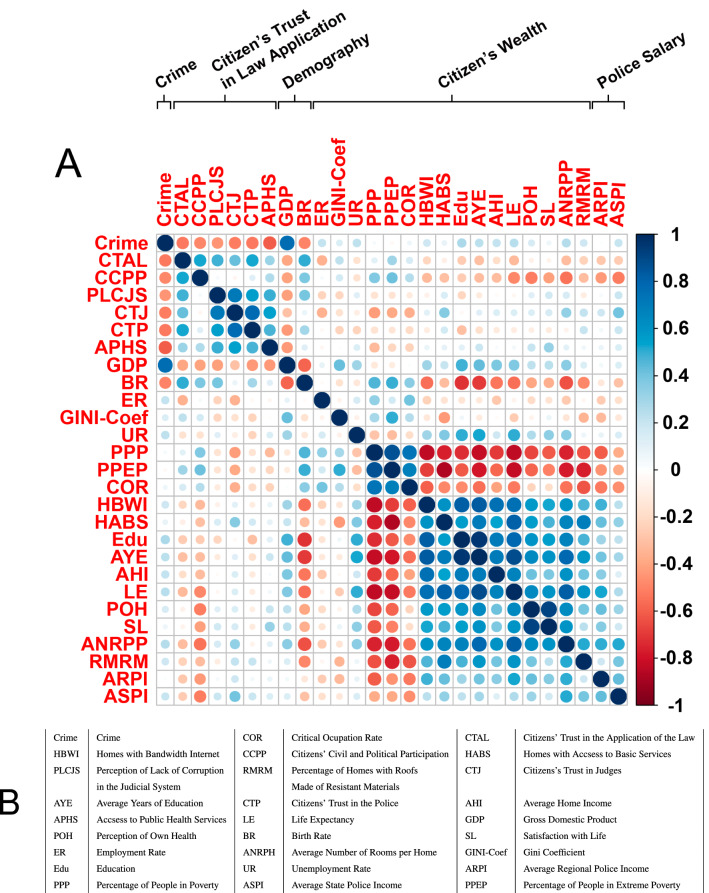
Correlation matrix between crime, trust in justice, and social welfare indicators. (**A**) The element in the first row and column (upper left corner) corresponds to the crime level (Crime). Note that crime is not significatively correlated with any of the social welfare indicators such as police salary, poverty, education, use of Internet, or family income. However, crime is highly anti-correlated (red dots, first row) with the parameters that measure citizens’ confidence in the police and justice system. Crime is also anti-correlated with access to public health services and birth rate. Interestingly, crime is positively correlated with Gross Domestic Product, a standard measure of wealth. (**B**) Meaning of the acronyms and abbreviations used in (**A**). The raw data to create this correlation matrix were taken from *Semáforo Delictivo* (crime)^26^ and the National Institute of Geography and Statistics (justice and welfare indicators)^37^.

To determine possible causes of crime in Mexico, we computed the Pearson’s correlation between crime incidence in each of the 32 Mexican States and 25 different indicators of social welfare in these States^37,38^. The resulting correlation matrix is shown in Fig. 2, in which the first element (first row and column) corresponds to crime. (See Supplementary Fig. S4 for an example of the type of data we are correlating). From Fig. 2 it can be observed that crime is not correlated with social welfare indicators such as family income, use of bandwidth Internet, unemployment or police salaries (see also Supplementary Fig. S4). However, crime is significantly anti-correlated with trust and confidence in police corporations and the justice system (red dots, first row and column): in the Mexican States where crime is higher, citizens’ trust in the police and justice system is considerably lower. Indeed, the *perception* that people have of corruption in the justice system and law-enforcement corporations is more strongly correlated with high levels of crime than low wages, education or unemployment. In the Discussion section we will elaborate on this result.

One possible reason for this anticorrelation may be due, indeed, to there being more police corruption in the regions affected with higher crime rates. Another possibility is that the perceived police and judicial corruption may lead citizens to believe that they can get away with crime unpunished. If this is the case however, it is obvious that not every citizen is a potential criminal. Broadly speaking, citizens can be grouped in three categories regarding the commission of crimes: (a) the ones that will never commit a crime because of their moral and social standards. (b) The ones that do not commit criminal acts because they are afraid of punishment. These citizens would likely commit a crime if the opportunity presents itself and they believe there is a way to avoid punishment. (c) The ones that have already committed crimes or readily will commit a crime (the criminals). Unfortunately, from the databases we analyzed it is not possible to know how many criminals are out there committing crimes. For instance, we know the number of reported stolen cars but we do not know how many times the same criminal steals a car in a month (or a week). However, in the Second National Survey on Constitutional Culture carried out by the Legal Research Institute of the National Autonomous University of Mexico, 19% of the people responded that they would be willing to break the law if they consider the law to be unfair, whereas 21% agreed that the problem is not to break the law, but to be caught^39^. In the same survey, about 10% of the people interviewed stated that they do not commit crimes for fear of being punished. (A similar survey was carried out by the Mexican Ministry of the Interior, with results analogous to the ones previously described^40^.) Breaking the law does not necessarily make anyone a criminal. But it is alarming that such a large proportion of people are willing to disobey the law if this suits their needs. For our model, we can consider 20% as the upper bound for the fraction of potential criminals in the society.

### Agent-based model

**Figure 3:**
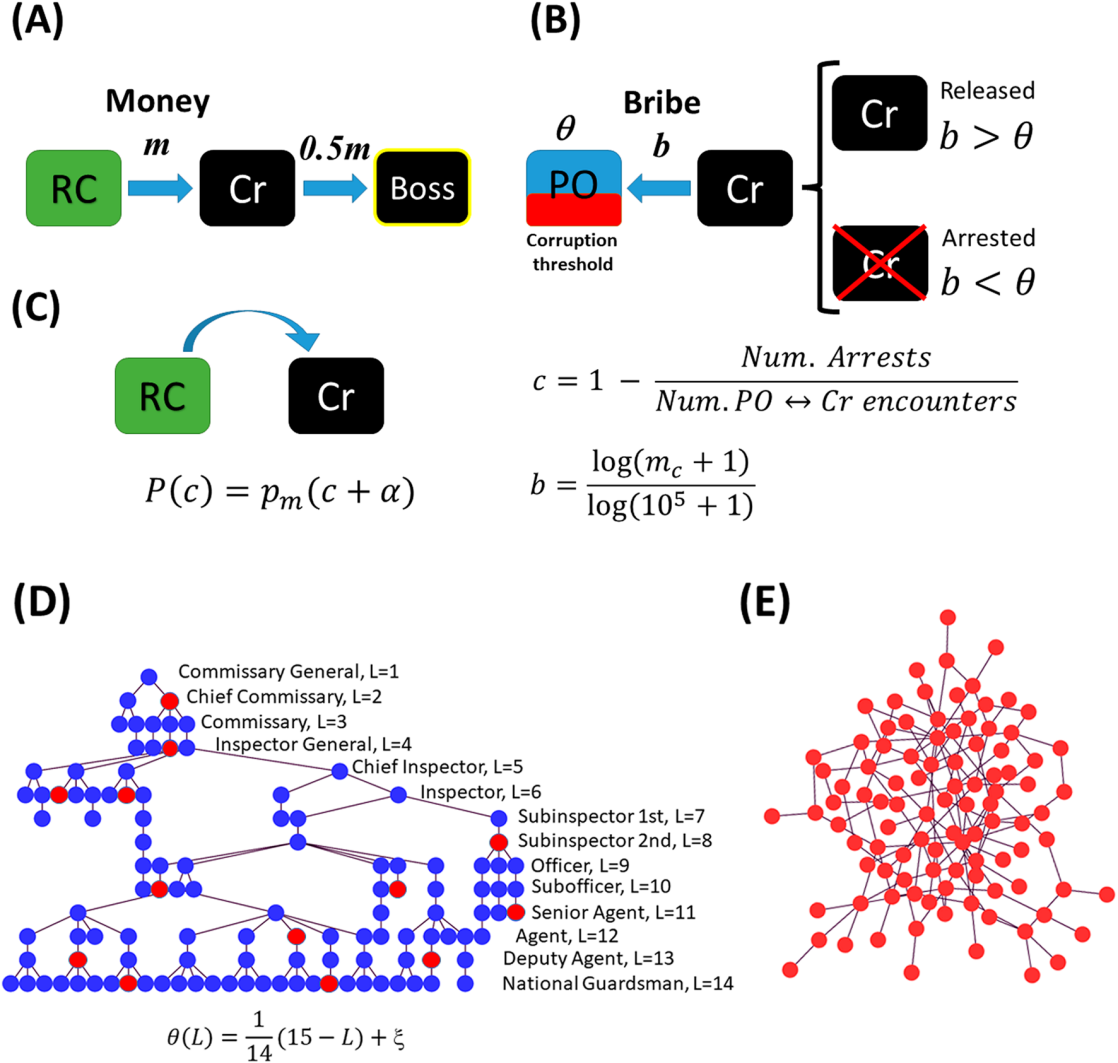
Agent-based model dynamics. (**A**) When a criminal (Cr) meets a regular citizen (RC), the criminal steels all the citizen’s money. The criminal then gives 50% of the stolen money to his boss (another criminal). (**B**) Police officers (PO) are characterized by a corruption threshold $\theta$. In a PO$\leftrightarrow$Cr encounter, the criminal offers a bribe *b* to the officer. If the bribe is larger than the officer’s corruption threshold, the criminal is released, otherwise he is arrested and disappears from the simulation. The corruption level *c* is a function of the ratio between the number of arrests and the total number of PO$\leftrightarrow$Cr encounters. (**C**) A RC has a probability *P*(*c*) of turning into a Cr. The turning probability *P*(*c*) is higher as the actual level of corruption level *c* increases. The parameter p_b_ = p_m_ $\alpha$ represents the increase perception of corruption and $p_m$ is a parameter that limits the maximum level of the citizen$\rightarrow$criminal turning probability. (**D**) Schematic representation of the police network. The 14 ranks and their corresponding levels are indicated. The corruption threshold $\theta$ of the corrupt officers (represented by red circles) is a linear function of the level *L*, plus a small noise $\xi$. (**E**) Schematic representation of the criminal network, which is constructed through a preferential attachment algorithm based on money rather than on the number of connections: when a regular citizen becomes a criminal, he has to work for a boss chosen with a probability proportional to the boss’ money.

The model consists of *N* agents (individuals) divided in three different groups: $N_p$ police officers, $N_c$ criminals, and $N_r = N-(N_p+N_c)$ regular citizens. The total number *N* of individuals and the number $N_p$ of police officers remain constant throughout the simulation, but the numbers $N_c(t)$ and $N_r(t)$ of criminals and regular citizens can change with time. At the beginning of the simulation every regular citizen is provided with a random amount of money $m\in (0,1)$ chosen with uniform probability. Crimes consist of criminals robbing the money from regular citizens (see Fig. 3A). (We generically refer to the criminal act as “robbery”, but in can be any act in which the criminal obtains money from the victim, such as extortion, kidnapping, or drug dealing.) At each time-step, which corresponds to a day, with probability $p_{rob}$ every criminal randomly selects a regular citizen and robs all his money (Fig. 3A). In this way, criminals accumulate money. From the databases that we analyzed, we can only know how many crimes, on average, are committed daily, but we do not know how many criminals are committing crimes at each time-step. For concreteness, we assume that, on average, every criminal commits a criminal act once a week. Therefore, at every time-step in our simulation, each criminal has a probability $p_{rob}=1/7$ of committing a crime on a randomly chosen citizen. Additionally, the number $N_c$ of criminals is bounded by $N_c \le 0.2N$, given the fact that approximately 20% of the population declared to be willing to break the law. These are the potential criminals in the society.

Most of the money-related crimes reported in Fig. 1 are committed by individuals that belong to mafias or cartels; these take a cut of the money stolen by a criminal in exchange for protection if the criminal is caught. To model this, we assume that criminals are organized in hierarchical networks in which each criminal works for a “boss” (see Fig. 3E). We start the simulation with $N_c(0)$ criminals at time $t=0$. Each of these initial criminals is born with a random amount of money $m_c$ chosen in the interval (0, 1) with uniform probability. We construct the network by connecting the criminals through a preferential attachment algorithm based on money instead of on the number of connections. Therefore, with probability $P(m_c^i)=C m_c^i$, each of the initial criminals will choose a boss within the set of criminals, where $m_c^i$ is the amount of money of the $i^{th}$-criminal and *C* is a normalization constant. The initial criminal network is thus already connected and looks like the one shown in Fig. 3E. As the simulation proceeds, when a regular citizen becomes a criminal, he connects to the network also choosing a boss with probability $P(m_c^i)$ using the same preferential attachment algorithm. This mechanism leads to a hierarchical criminal network where richer criminals will eventually have more people working for them, which is consistent with empirical observations^41^. In the simulations presented here, the percentage of the loot that a criminal shares with his boss for each crime committed is 50%, but the results do not change qualitatively for percentages between 30% and 70%. After the robbery, the robbed citizen is provided again with a new random amount of money *m* uniformly distributed in the interval (0,1).

Regarding the police corporation, according to the National Census of Federal Public Security 2021 reported by INEGI^42^, and the report 2022 Mexico Military Strength^43^, there are about $6\times 10^{5}$ active elements in the law enforcement corporations in Mexico, including Police, National Guard, Army, Marines and Air Force. Not all of these elements are active at the same time. Assuming that only half of the members of the law enforcement corporations are active simultaneously, and taking into account that the economically active population of Mexico is about 57 million, then the number $N_p$ of effective “police officers” combating crime is between 0.005*N* and 0.01*N*. (In this work we generically refer as “police officer” to any member of the law-enforcement corporations officially in charge of combating crime, whether a state policeman, a marine or a soldier. No offense intended to anyone.) In all the simulations presented here we fixed $N_p = 0.01N$ to illustrate the dynamics of crime with the maximum, but realistic, number of police officers.

At every time-step, each police officer has a probability $p_{cap}=1/7$ of capturing a randomly chosen criminal. In a criminal-officer encounter, we distinguish between “capturing” and “arresting”. Capturing just means a police-criminal encounter in which the criminal offers a bribe to the police officer. If the officer is corrupt and the bribe is high enough, the criminal is released. Otherwise, if the police officer is honest or the bribe is not high enough, the criminal will be arrested (disappearing from the simulation). To implement this dynamics, police officers are assigned a corruption threshold $\theta$. Honest officers have a threshold $\theta =\infty$ (they will never accept a bribe), whereas a corrupt officer has a threshold $\theta \in [0,1]$ that depends on his rank. When a police officer encounters a criminal (Fig. 3B), one of three things can happen:

- If the police officer is honest ($\theta =\infty$), then the criminal is arrested and disappears from the simulation, contributing to reduce the crime incidence.
- If the police officer is corrupt ($0\le \theta \le 1$), he will accept a bribe *b* from the criminal (or from the criminal’s boss) if the bribe is higher than the officer’s corruption threshold ($b\ge \theta )$. In that case the policeman releases the criminal.
- If the officer is corrupt and the bribe offered to him is lower than his corruption threshold ($b<\theta$), the criminal is arrested and disappears from the simulation.

The maximum bribe a criminal can pay is the total amount of money $m_c$ he has accumulated. If the criminal does not have enough money to pay the bribe, with probability $p_{resc} = 0.2$ the criminal’s boss will pay it as long as he has enough money to surpass the officer’s corruption threshold, thus rescuing the subordinate criminal from being arrested. This rescuing mechanism provides a clear advantage for criminals to belong to a criminal network. When neither the criminal nor his boss have enough money to pay the bribe, the captured criminal is arrested and disappears from the simulation. To keep the population size constant, every criminal that is arrested is replaced by a regular citizen. Each time a criminal or his boss pay a bribe, they lose all their money. These criminals will gradually accumulate money again by robbing regular citizens. It is important to emphasize that paying bribes does not change the topology of the criminal network. Criminals that pay bribes are left with no money, but they are still part of the network with the same connections. Only arrests can change the network topology because arrested criminals are removed from the network, which also removes all their connections.

To assign the corruption threshold to every police officer, we take into account the hierarchical structure of the Mexican National Guard, which has 14 levels (or ranks), being the Commissioner General the policeman with the highest rank (level $L=1$) and the National Guardsman the one with the lowest rank (level $L=14$). Figure 3D shows a schematic representation of the police network, with honest and corrupt officers represented by blue and red circles, respectively. The corruption threshold $\theta (L)$ of a corrupt officer with level *L* is a decreasing function of *L*. We tried different decreasing function with qualitatively the same results. In what follows, we will use the linear function

1 $$\begin{aligned} \theta (L)=\frac{1}{14}\left( -L+15\right) +\xi , \end{aligned}$$

where $\xi$ is a small random number in the interval $\xi \in [-0.01,0.01]$ whose purpose is to introduce variability among officers within the same rank. With the function given in Eq. (1), the higher the rank of the officer, the higher his corruption threshold and hence, the higher the bribe the criminal has to offer to be released. Only very rich criminals can bribe high-rank officers. As indicated in Fig. 3D, a fraction $F_c$ of police officers, randomly chosen from anywhere in the police network, are corrupt. It is important to stress the fact that honest cops always have a corruption threshold $\theta =\infty$, whereas corrupt officers have $0\le \theta \le 1$. The fraction $F_c$ of corrupt officers in the police network (or equivalently, the fraction $F_h=1-F_c$ of honest officers) is one of the most important parameters of the model.

To simulate the corruption dynamics within the police corporation in our model, when a corrupt high-rank police officer commits his first corruption act (accepts a bribe for the first time), all the corrupt police officers under his commands will half their corruption threshold. In this way, corruption within the police corporation does not randomly occur in isolated officers, but it occurs in hierarchical branches of command. This is a strong assumption that can be considered as an upper bound to the actual corruption patterns occurring within the police corporation.

Since the corruption threshold $\theta$ of each corrupt officer is normalized in the interval [0, 1], the bribe *b* a criminal can offer must also fall in the same interval. Therefore, we define the normalized bribe as

2 $$\begin{aligned} b = \frac{\ln (m_c+1)}{\ln (10^5+1)}, \end{aligned}$$

where $m_c$ is the total accumulated money of the captured criminal offering the bribe. In this way, both corruption thresholds and bribes are normalized in the same interval. However, note that if the criminal’s money exceeds $10^5$, then the bribe *b* will be larger than 1. In this case, corrupt officers will always accept the bribe regardless of their rank. The quantity $10^5$ (in arbitrary units) is intended to represent a great amount of money. The purpose of the logarithm in Eq. (2) is to increase the rate at which criminals accumulate capacity to bribe policemen as they begin to commit crimes, and decrease this rate when the accumulated money $m_c$ approaches $10^5$, allowing the system to reach a steady state faster than if the bribes were, say, a linear function of the accumulated money $m_c$ (see Supplementary Fig. S5).

Finally, there is a turning probability *P* that a regular citizen becomes a criminal (Fig. 3C). This probability depends on the level of corruption in the society, which is defined as follows. Let *M*(*t*) and *A*(*t*) be the number of captures (police-criminal encounters) at time *t*, and the number of these captures that resulted in arrest, respectively. We define the corruption level as $c(t)=1-A(t)/M(t)$. If all the captures resulted in arrests then $c=0$, whereas $c=1$ when in all these encounters the police officers were bribed and let the criminals escape. The turning probability *P*(*t*) that a regular citizen becomes a criminal is then given by

3 $$\begin{aligned} P(t) = \left( c(t) + \alpha \right) p_m , \end{aligned}$$

where $p_m$ is the maximum turning probability associated to the corruption level *c*(*t*), and $\alpha$ is the contribution due to people’s increased perception of corruption. Increasing the value of $\alpha$ would correspond to increasing people’s perception of corruption beyond its actual value *c*(*t*), which in turn increases the turning probability *P*(*t*) that a regular citizen becomes a criminal. It is understood in Eq. (3) that if *P*(*t*) exceeds 1, then we set $P(t)=1$. The turning probability *P*(*t*) changes with the level of corruption *c*(*t*) and reaches a maximum value $p_m(1+\alpha )$.

It is important to note that the turning probability *P*(*t*) only applies to the 20% of regular citizens that represent potential criminals (the ones willing to break the law), which are randomly chosen from anywhere in the system with uniform probability. Once a regular citizen becomes a criminal, he will continue being a criminal in the simulation until he is arrested. In all the results presented here, we start the simulation with an initial number $N_c=0.1N$ of criminals. Throughout the simulation this number can grow as more regular citizens become criminals, but it will never exceed 20% of the total population. $N_c$ can also decrease as honest cops arrest more criminals. We will see that the the turning probability *P*(*t*), and thus the parameters $p_m$ and $\alpha$, play a very important role in the dynamics of the model.

To determine the level of crime in the system we define the instantaneous order parameter as $\psi (t)=N_v(t)/N$, where $N_v(t)$ is the number of victims of crime at time *t* and *N* the total number of agents in the system. We let the simulation run until $\psi (t)$ reaches a stationary value (see Supplementary Fig. S6). Then, we compute the stationary value $\psi$ of the order parameter as

4 $$\begin{aligned} \psi = \frac{1}{T_m-T_0}\sum _{t = T_0}^{T_m} \psi (t), \end{aligned}$$

where $T_0$ is a transient time long enough to let the system reach the stationary state, and $T_m$ the maximum computing time. If $\psi \approx 0$ then the system reaches a stationary state where there is no crime, whereas if $\psi \approx 0.8$ the stationary state is such that almost all regular citizens have been victims of crime. Note that the saturation value of the order parameter is $\psi \approx 0.8$ and not $\psi =1$. This is because in the worst scenario 20% of the citizens have become criminals while the other 80% (the victims) will never become criminals.

### Model dynamics

**Figure 4:**
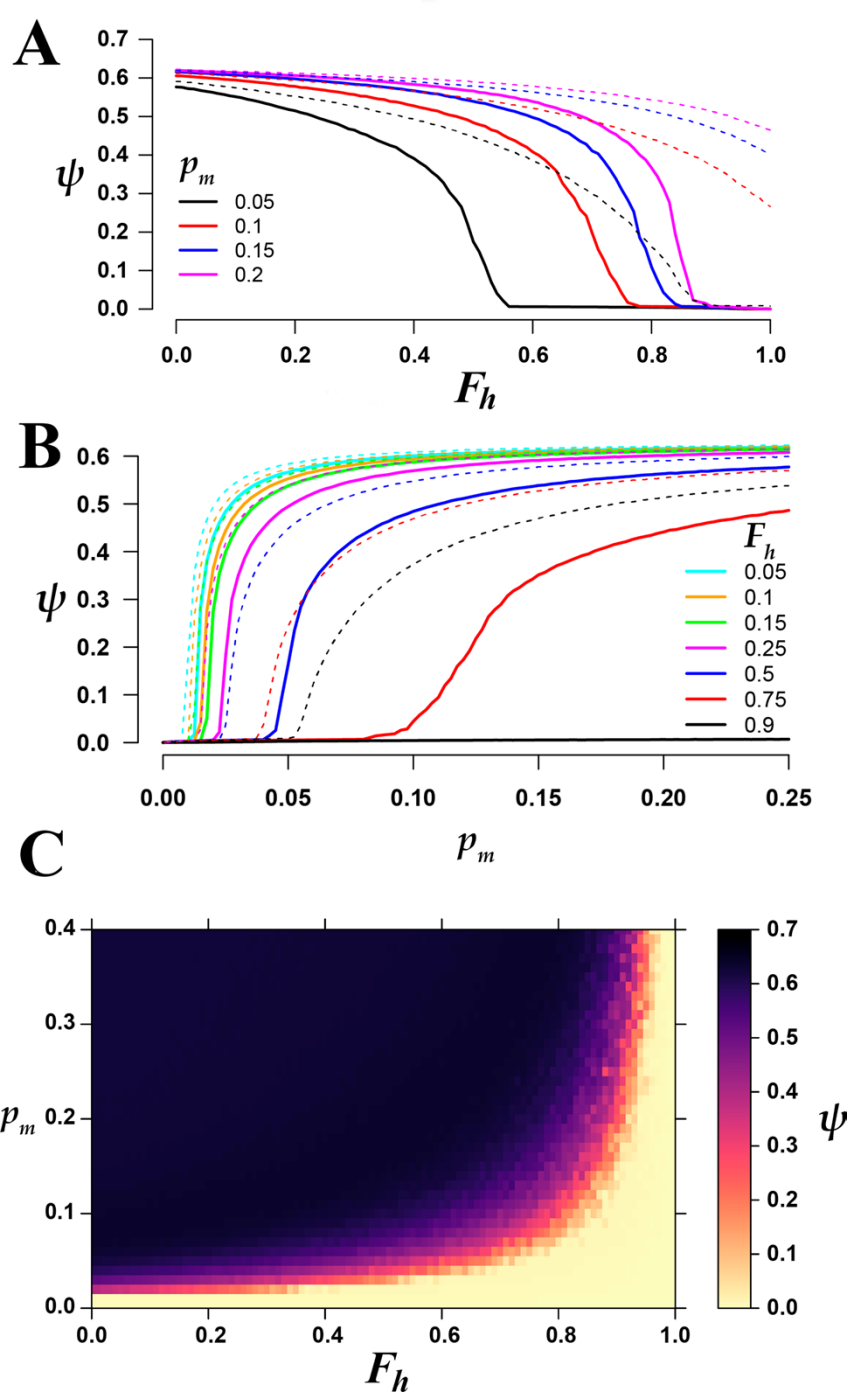
Phase transition. (**A**) Phase transition of the crime incidence $\psi$ as a function of the fraction $F_h=1-F_c$ of honest police officers ($F_c$ is the fraction of corrupt officers). The solid curves correspond to different values of the maximum turning probability $p_m$ with no inflated corruption perception ($\alpha =0$). The dashed-broken lines correspons to the same values of $p_m$, but with an increased perception of corruption $\alpha =0.2$. (**B**) Phase transition of $\psi$ as a function of the turning probability $p_m$ for different values of $F_h$ and $\alpha =0$ (solid lines), and for $\alpha =0.2$ (dashed-broken lines). (**C**) Heat map of the phase transition as driven by both $F_h$ and $p_m$ for $\alpha =0$. Note that the phase transition is remarkably sensitive to changes in $p_m$ as compared to changes in $F_h$. All the simulations were performed for systems with $N=10^5$ agents, 1% of which were policemen and 10% criminals in the initial condition. Throughout the simulation the number of criminals may vary but the number of police officers remains fixed.

Figure 4A shows the existence of a phase transition in the order parameter $\psi$ (crime incidence) as a function of the fraction $F_h$ of honest police officers for different values of the maximum turning probability $p_m$ when there is no increased perception of corruption (namely, for $\alpha =0$, solid lines). This phase transition seems to be of second order, as the variance (susceptibility) of the order parameter shows a clear peak at the value at which the phase transition occurs (see Supplementary Fig. S7). For a given value of $p_m$, as $F_h$ increases the system transits from a saturated state with maximum crime to a state with no crime. The critical value $F_h^*$ at which the phase transition occurs increases as $p_m$ becomes larger. This is not unexpected, as the more people become criminals, the more honest police officers are needed to control crime. Figure 4A also shows the dramatic effect of a non-zero increased perception of corruption $\alpha =0.2$ (dashed-broken lines), for which the phase transition is considerably shifted to larger values of $F_h^*$ and completely destroyed for $p_m\ge 0.1$, leaving the system in a state of permanent criminality regardless of the fraction $F_h$ of honest police officers.

Figure 4B shows another phase transition driven by $p_m$ for fixed values of $F_h$ and $\alpha =0$ (solid lines). This phase transition, which appears to be of second order as well, (see Supplementary Fig. S8), is considerably more sensitive to changes in $p_m$ than in $F_h$, as for low values of $F_h$ (less than 0.5) the phase transition driven by $p_m$ becomes almost discontinuous. Therefore, when there are relatively many corrupt officers, even a small increase in the turning probability $p_m$ can lead to crime saturation. Again, for a non-zero increased perception of corruption $\alpha =0.2$, the phase transition driven by $p_m$ is enhanced, leading to a system with high levels of crime (dashed-broken lines). Note in particular that for $F_h=0.9$ and $\alpha = 0$ there is no crime (black solid line at $\psi =0$), whereas for the same value of $F_h$ and an increased perception of corruption $\alpha =0.2$, the phase transition occurs at $p_m\approx 0.05$ (black dashed line). This illustrates the dramatic effect that an inflated perception of corruption can have on the level of crime in the society. Figure 4C shows the two-parameter phase transition driven by both $F_h$ and $p_m$ for $\alpha =0$. All the simulations reported in Fig. 4 were performed with $N=10^5$ individuals, of which $N_{p}=1000$ were police officers and an initial population of $N_c=10,000$ criminals. Note that $N_{p}$ is fixed throughout the simulation whereas $N_c$ can vary depending on the level of corruption.

**Figure 5:**
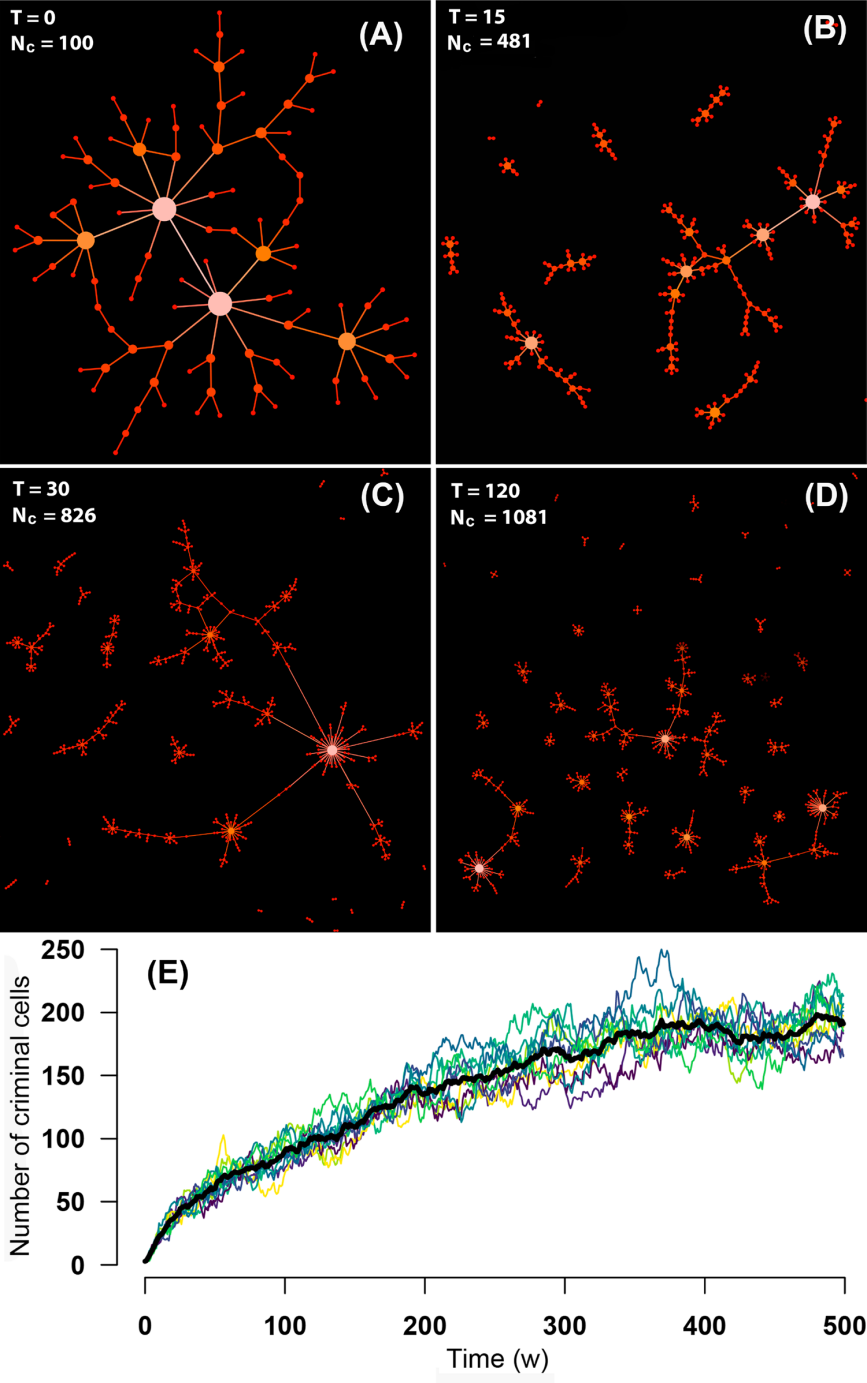
Evolution of the criminal network. (**A**) Initially, the criminal network is fully connected and exhibits a scale-free-like topology (although the network in this example is too small –$N_c = 100$– to actually define a power-law degree distribution; however, the existence of hubs is clear). (**B**)–(**D**) As the simulation proceeds and police officers capture criminals, the criminal network fractures into independent subnetworks (*criminal cells*). (**E**) The total number of criminals and independent cells increase despite the capturing of criminals by police officers. Panels (**A**)–(**D**) were computed with a small system ($N=10,000$, $N_p = 100$, and an initial number of criminals $N_c=100$) for illustrative purposes. Panel (**E**) was computed for systems with $N=10^5$ individuals, $N_p = 10^3$ police officers, and an initial population $N_c=0.1N$ of criminals. Different curves in color correspond to 10 distinct realizations. The bold-black curve is the average of these realizations.

Interestingly, as police officers capture criminals, the criminal network undergoes dramatic changes. For illustrative purposes, Fig. 5A shows the initial structure of the criminal network for a relatively small system with $N=10,000$, $N_{p}=100$ and an initial population of criminals $N_c=100$. Other parameters were chosen to preserve a high level of crime in the population ($\psi \sim 0.6$), so that the criminal network does not disappear over time. As the simulation proceeds, the criminal network fractures into an increasing number of independent subnetworks (or criminal *cells*, Fig. 5B–E). Despite this fracturing, which has actually been observed in the structure of criminal networks in Mexico^19–24^, both the total number of criminals and their accumulated money increase with time (see Supplementary Fig. S9).

A strategy that has been widely used by authorities to fight organized crime is the seizure of criminal money, assets and drug shipments^44,45^. To simulate this strategy in our model, we let crime evolve and stabilize for 2700 time steps (300 weeks). At that moment (which we will refer to as the *blocking time*), we eliminate the money of 80% of the richest criminals in the network. Without that money, these criminals will not have the means to bribe police officers and will be arrested (disappear from the simulation) when captured. We maintained the fraction of honest officers $F_h=0.5$ and the value of corruption perception $\alpha =0$ to analyze this strategy in a scenario with high levels of police corruption.

**Figure 6:**
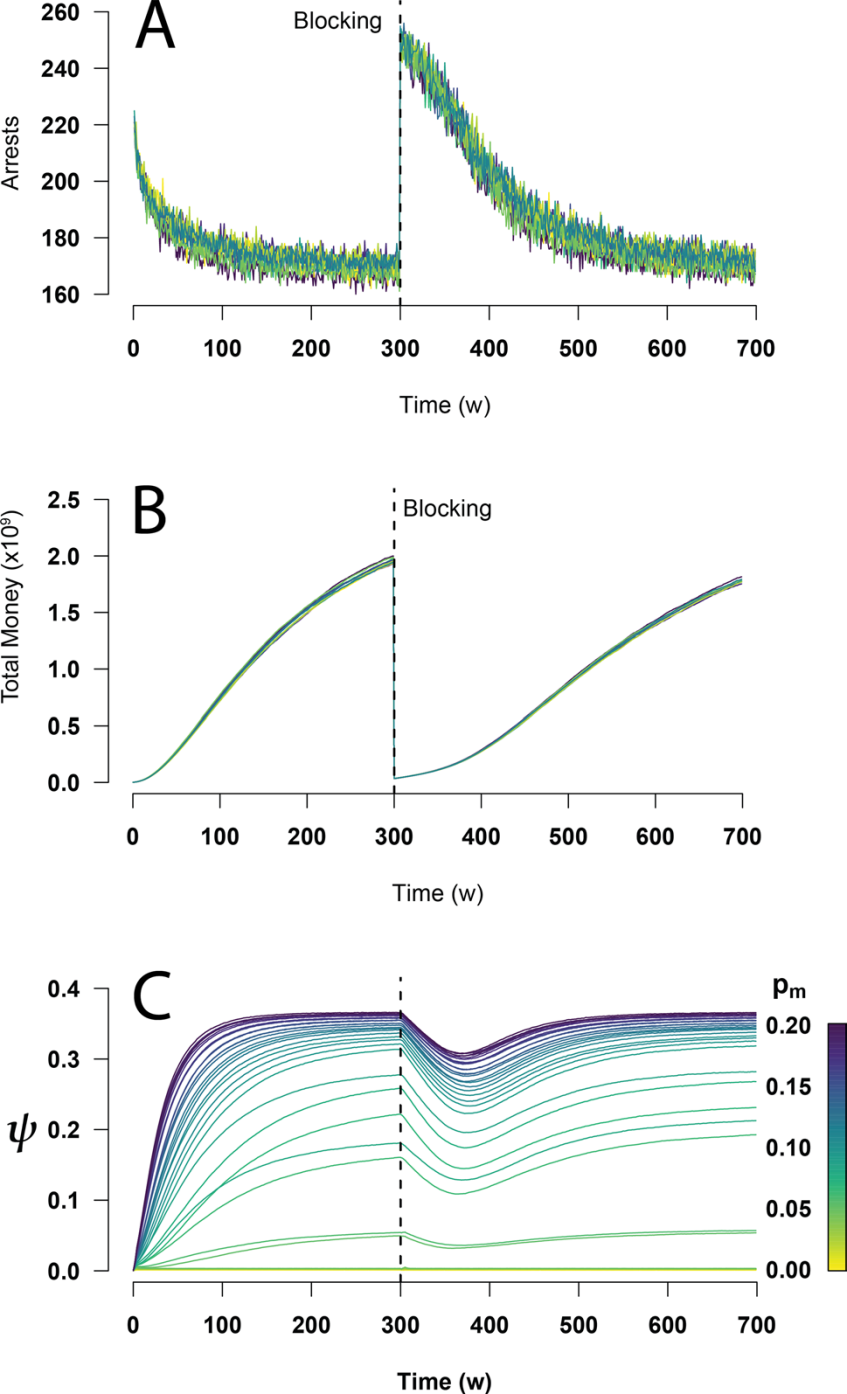
Effect of money seizure. After 2700 time steps (300 weeks) the money of 80% of the richest criminals in the criminal network is seized (or blocked), indicated by the vertical dashed line in all the plots. (**A**) Number of arrests as a function of time. Immediately after the money has been blocked, the arrests sharply increase since criminals do not have enough money to bribe police officers and get away. (**B**) The total amount of money in the criminal network abruptly decreases after the blocking. However, both the number of arrests and the amount of total money rapidly return to the stationary values observed before the blocking. (**C**) Surprisingly, contrary to what happens with the number of arrests and the accumulated criminal money, the crime incidence $\psi$ is barely affected by the money blocking. The simulations for this figure were carried out for systems with $N=10^5$ and $\alpha =0$.

Although simultaneously blocking the money of 80% of the richest criminals in the society is not realistic, we want to analyze the effect that such strategy has on the level of crime in an extreme case. Figure 6 shows that immediately after blocking (or confiscating) the money, the number of arrests sharply increases as the majority of the criminals do not have enough money to bribe police officers (Fig. 6A). However, since in this case the corruption *c*(*t*) is high, captured criminals are rapidly replaced by regular citizens who become criminals. Under such circumstances, although most of the money in the criminal network has been confiscated (Fig. 6B), the crime incidence is almost unaffected in the long run, as Fig. 6C illustrates. Therefore, if the fraction $F_c$ of corrupt police officers, the maximum turning probability $p_m$, and the corruption threshold $\theta$ stay the same, the level of crime does not remain at low values under money confiscation, but rapidly returns to the stationary value it had before the blocking time.

## Discussion

It has been assumed by several politicians, policy makers, and scholars around the world that poverty and inequality are major causes of criminal behaviour^46–48^. While this may be true for certain countries and populations, this does not seem to be true for Mexico, as Fig. 2 shows. We are aware that this result flies in the face of a great body of work suggesting a strong positive correlation between poverty and inequality on the one hand, and criminality on the other hand^30–32,49–53^. Nonetheless, the cause-effect relationship between poverty and criminal behavior is still controversial, at least for some regions^54,55^. The correlations presented in Fig. 2 indicate that, while poverty and inequality may be important for the emergence of violent crime in Mexico, they may not be the main causes, and while a positive correlation between two events, does not imply a causal relationship, *a lack* of correlation does imply a lack of causality between them. Figure 2 clearly reports that, to the best of the current data available, there is essentially no correlation between the level of crime and any indicator of social welfare. This is consistent with the fact that organized crime, (particularly related to drug trafficking, kidnapping, extortion, and other ramifications), in Mexico did not emerge from the poorest Mexican states (as measured by the GDP, such as Guerrero, Oaxaca, or Chiapas). By contrast, organized crime in Mexico started in rich states such as Sinaloa, Jalisco and Durango, which are historically characterized by having very important fishing, agricultural, livestock and/or technological industries^24^. By *a priori* considering poverty as a main source of crime, policy makers can make decisions that are not necessarily effective in reducing crime.

We found that crime incidence is significantly more anti-correlated with people’s trust in law-enforcement institutions and the justice system than with welfare indicators. As discussed earlier, this may be due to the fact that there may actually be more corruption in the regions with higher crime rates, but another possible explanation is that, when the State and the law-enforcement institutions have lost legitimacy (justifiably or not), regular citizens who may be potential criminals think that they can escape justice, cross the line and become criminals, thinking that there will be no consequences.

Although previous models have analyzed organized crime using complex network tools^56–61^, to our knowledge, this is the first time in which a mathematical/computational agent based model of organized crime incorporates a feedback between the corruption (and its perception) of police corporations and the incidence of crime in a society. We do this by first introducing a fixed fraction $F_c$ of corrupt police officers who release a criminal if the bribe is high enough, and second by increasing the probability $p_m$ that a regular citizen becomes a criminal depending on the level of corruption, either real and/or perceived. There is also a parameter $\alpha$ which represents the *excess perception of corruption * in the society. The two parameters $F_c$ and $p_m$ drive the system through a phase transition from a regime with essentially no crime, $\psi \approx 0$, to a regime in which the crime rate quickly saturates, $\psi \approx 0.8$ for $\alpha =0$, namely, when the perception of corruption is accurate. Computations of the variance (susceptibility) of the order parameter suggest that this phase transition is of second order, but a more detailed analysis is necessary to determine the detailed properties of the phase transition. The important point, however, is the existence of a transition from a regime with low criminality to a regime with high crime incidence. Remarkably, in the case where there is even a relatively small increase in the perception of corruption ($\alpha = 0.2$) above its real level, the phase transition is mostly destroyed. Another important consequence of the existence of a phase transition is that the lower and upper bounds for the level of criminality that are observed in Mexico (1.5% and 15%, respectively), may be close to the critical point in both $F_h$ and $p_m$, as Supplementary Figs. S7 and S8 illustrate. This suggests that, even assuming the upper bound of 15% crime incidence, which corresponds to a dark figure of 90%, by slightly reducing the probability that regular citizens become criminals the system can cross the critical point to the low-crime phase. However, for this to happen, the right decisions should be made to restore the legitimacy of law-enforcement corporations.

We do not claim that the lack of trust in the police and the justice system is the sole cause of criminality. We do not argue either that our model realistically captures the critical values $F_h^*$ and $p_m^*$ at which the phase transition occurs. Our main purpose in this work is to show the drastic effect that an inflated perception of corruption $\alpha$ has on the levels of crime: a high perception of corruption leads citizens to disrespect authorities and behave out of the law, significantly increasing the level of crime. It is worth emphasizing that even a value of the inflated perceived corruption as small as $\alpha = 0.2$ considerably increases the level of crime, leaving the system in a permanent state of criminality for most values of the other relevant parameters, as Fig. 4 shows.

In light of these results, to reduce crime in a society it is not enough to eliminate corruption from law-enforcement corporations. It is also important to instill respect in citizens for these institutions and the justice system, since crime can propagate across the society even when the majority of police officers behave honestly. If the inflated perception of corruption citizens have of the law-enforcement corporations does not change, it matters neither how many organized-crime kingpins are arrested nor how much of their assets are seized, crime will never end. Indeed, our results also corroborate that, despite the fact that criminal kingpins have been captured and their assets seized, organized crime does not only not decrease, but actually grows, as has been demonstrated throughout the years by direct and indirect evidence such as the increasing number of captured criminals^22,23^, seized drug shipments^45^, the number of people who die from drug abuse^62^, as well as an increase in the resilience of the criminal network^25^.

We believe that the results presented in this work should be considered by policy makers in developing countries, like Mexico, in which the widespread and disproportionately high perception of police corruption among citizens leads to a lack of respect for the institutions and those in charge of maintaining order in the society (judges, prosecutors, marines, soldiers, police officers, etc.).

Finally, this work can be extended in multiple directions. For example, we have shown that under certain circumstances, capturing criminals does not reduce crime, but only fractures the criminal network down into independent cells. This result is corroborated by empirical evidence and examples abound^19–25^. (When the leader of the Gulf Cartel was arrested, the cartel fractured into two opposite groups: the Gulf Cartel and the Zeta Cartel. When the leaders of the Zeta Cartel were captured or neutralized in combat, it fractured into several other cartels: Caballeros Templarios, La Familia Michoacana, Guerreros Unidos, etc., all of them fighting for territory and dominance.) Typically, when a big criminal organization is fractured, the independent cells that emerge start fighting against each other. We have not implemented these conflicts between criminals in our simulations and it would be interesting to analyze the consequences of such behavior. It would also be worth implementing a feedback between the perception of corruption in the society and the fraction of corrupt police officers. Such feedback was implemented for the fraction of criminals, but as the Labelling Theory predicts, the larger the perception of corruption in the society the more likely it is that some police officers become corrupt. Another aspect that we have not considered is the fact that police corporations usually fight against corruption within their ranks, ceasing or arresting corrupt officers. Honest police officers fighting corrupt police officers could give a quite different dynamics. Analyses in these directions are currently under study.

## Supplementary Information

Supplementary Information.

## Data Availability

The data reported in Fig. 1A, Supplementary Figs. S2A and S3, are publicly available in the *Semáforo Delictivo* repository, http://www.semaforo.com.mx/. (To download the number of crimes in each Mexican state one has to go to the menu “Tabla” of the main page, and select the state and type of crime at the bottom of the page. A table with the information is displayed.) To compute the crime incidence reported in Fig.1B we divided the total number of crimes in each Mexican state by its population. The data about the total population in each Mexican state are publicly available in the *National Institute of Geography and Statistics (INEGI)* repository, https://www.inegi.org.mx/app/tabulados/interactivos/?pxq=Poblacion_Poblacion_01_e60cd8cf-927f-4b94-823e-972457a12d4b&idrt=123&opc=t. The data regarding welfare indicators used to generate Fig.2 are publicly available in the *INEGI* repository, https://www.inegi.org.mx/app/bienestar/. The data used to generate Fig. S1 are available in the Semáforo Delictivo repository, the *Department of Public Security* repository (https://www.gob.mx/sesnsp/articulos/incidencia-delictiva?idiom=es), and the *INEGI* repository (https://www.inegi.org.mx/temas/incidencia/). The data reported in Supplementary Fig. S2B are publicly available in the *INEGI* repository, https://www.inegi.org.mx/temas/empleo/. The data used to generate Supplementary Fig. S4 are publicly available in the *DataMexico* repository https://datamexico.org/es/profile/occupation/policias-y-agentes-de-transito?employSelector1=salaryOption, and the *INEGI* repositories https://www.inegi.org.mx/programas/pibent/2013/#Datos_abiertos and https://www.inegi.org.mx/app/tabulados/default.html?nc=624. The data reported in all other figures of this work were generated through the agent-based model simulation. The Java code of this model is available from the corresponding author on reasonable request.
