# Supplementary material for: Modeling the role of police corruption in the reduction of organized crime: Mexico as a case study
Source: Sci Rep. 2022 Nov 10;12:19233. doi: 10.1038/s41598-022-23630-x (PMC9649790; doi:10.1038/s41598-022-23630-x)
Supplement: Supplementary file 1 — Supplementary Information. [file 41598_2022_23630_MOESM1_ESM.pdf]

Supplementary Information for Modeling the role of police corruption in the reduction of organized crime: Mexico as a case study

Andrés Aldana[1,2,+], Hernán Larralde[3,+], and Maximino Aldana[2,3,+,*]

[1]Instituto de Biotecnología, Universidad Nacional Autónoma de México. Cuernavaca, Morelos, México.
[2]Centro de Ciencias de la Complejidad, Universidad Nacional Autónoma de México. Ciudad de México, México.
[3]Instituto de Ciencias Físicas, Universidad Nacional Autónoma de México. Cuernavaca, Morelos, México.
[*]max@icf.unam.mx
[+]All authors contributed equally to this work

ABSTRACT

We present Supplementary Figures to further support the results and discussion of the Main Text.

Figure S1. Consistency of three different databases: *Semáforo Delictivo*, *National Institute of Statistics and Geography*, and *Department of Public Security*.
Figure S2. Total number of high-impact crimes in Mexico per year since 2016 to 2021, and the number of economically active people in Mexico in the same years.
Figure S3. Different types of crime in Mexico from 2016 to 2021.
Figure S4. Example of correlations between welfare indicators and crime in Mexico.
Figure S5. Graphical justification to use a logarithmic function to compute the capacity that criminals have to bribe police officers.
Figure S6. Examples of the temporal evolution of the order parameter ψ, which represents the crime incidence.
Figure S7. Phase transition with respect to the fraction F_h of honest police officers. The variance of the order parameter suggests that the phase transition is of second order.
Figure S8. Phase transition with respect to the probability p_m that a regular citizen becomes a criminal. The variance of the order parameter suggests that the phase transition is of second order.
Figure S9. Although many criminals are arrested and the criminal network is fractured, the total money available to the entire criminal network increases throughout time.

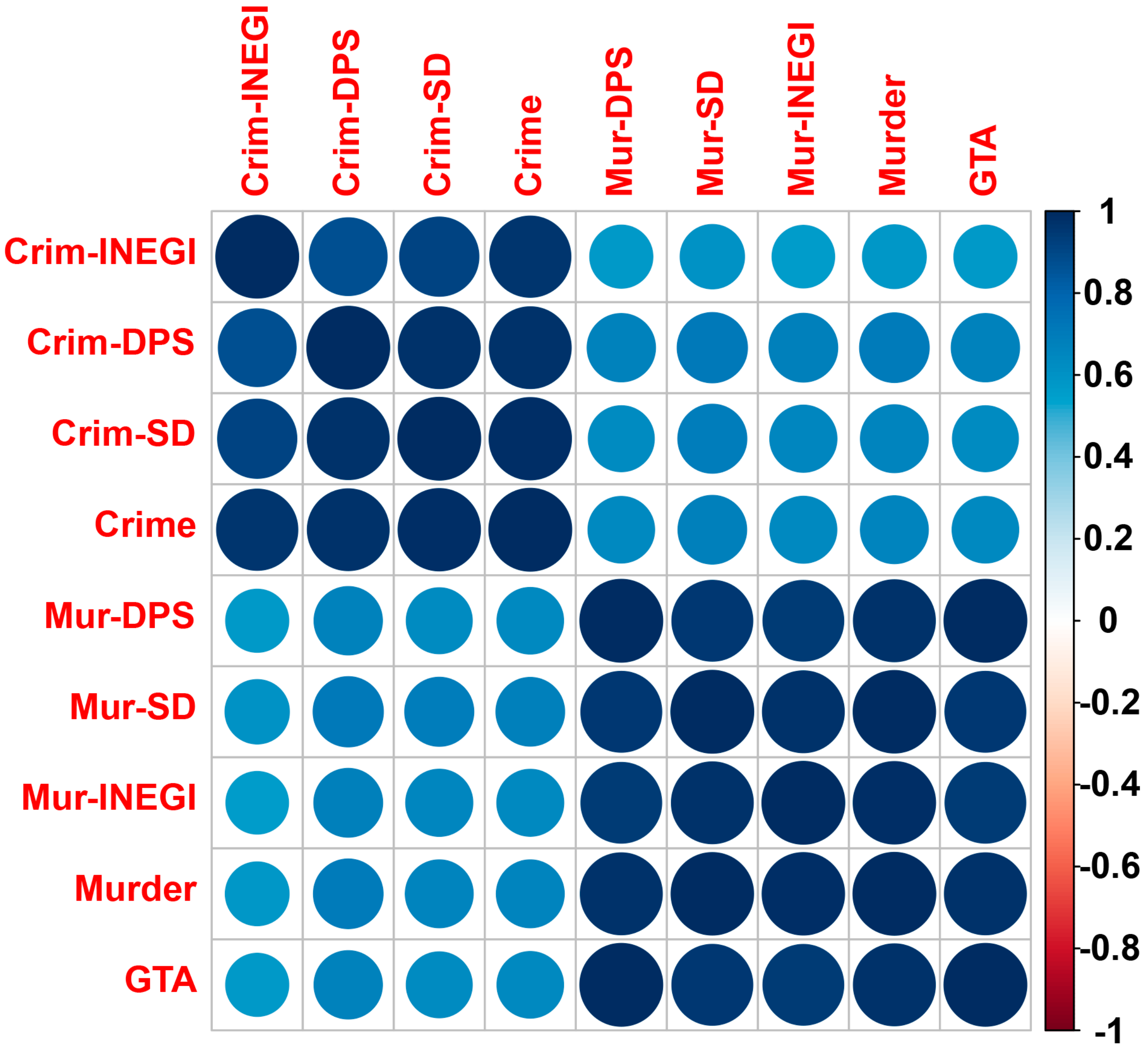

Figure S1. Comparison of different databases. Correlation matrix of the crime data reported in three different databases: Semáforo Delictivo (SD), the National Institute of Statistics and Geography (INEGI), and the Department of Public Security (DPS). The first four rows and columns correspond to all types of crime reported in Fig.1 of the main text. Rows and columns from 5th to 8th correspond to murder only, and the last column and row correspond to car theft (GTA). We selected murder and car theft because these are two of the best-reported crimes and hence do not appreciably contribute to the dark figure. It is clear from this figure that the three databases are highly correlated.

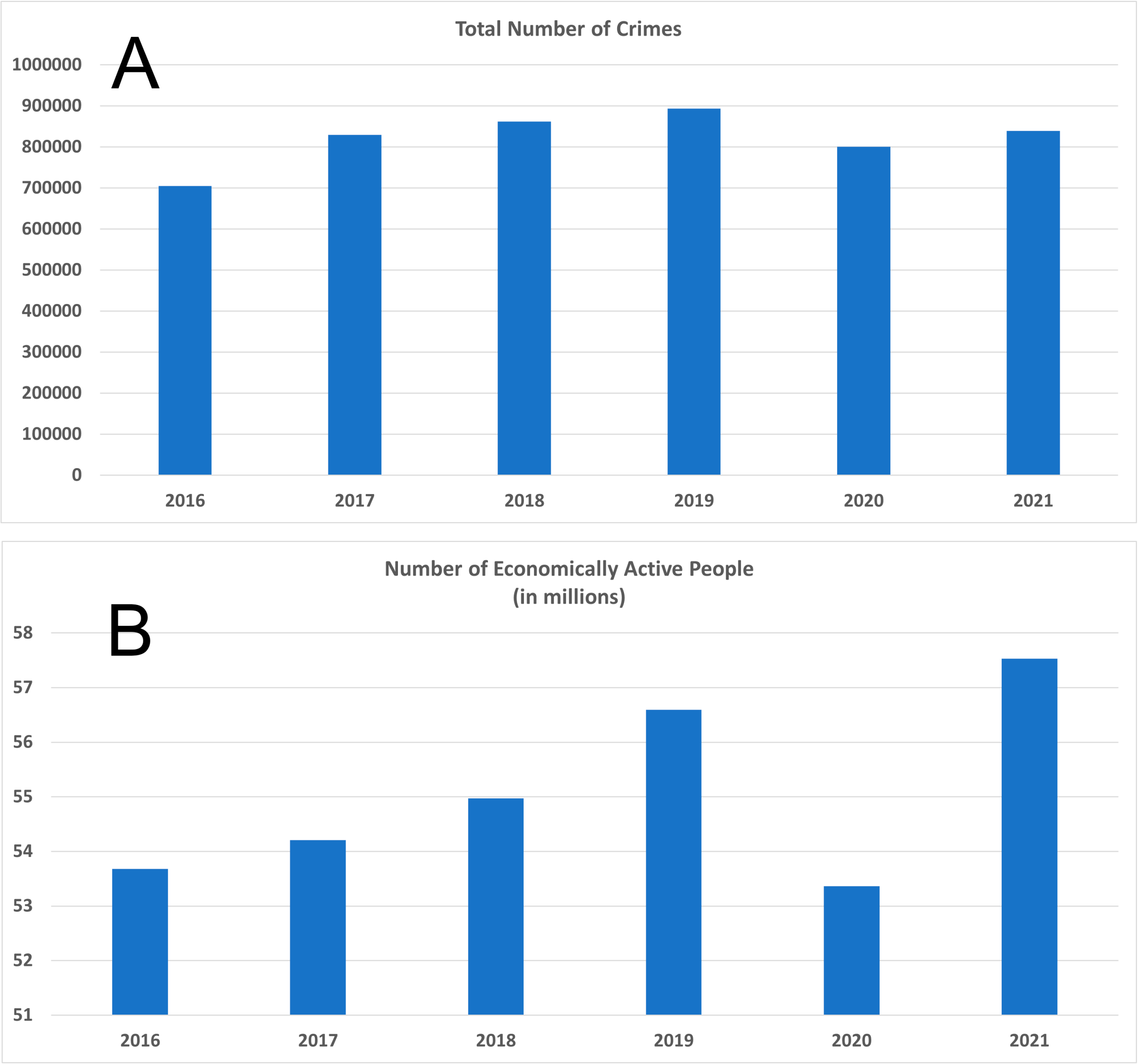

Figure S2. **(A)** Total number of high-impact crimes per year in Mexico from 2016 to 2021. Data obtained from Semáforo Delictivo (http://www.semaforo.com.mx/). **(B)** The number of Economically Active People per year in Mexico from 2016 to 2021. Note the effect of the COVID-19 pandemic in 2020. Data taken from INEGI (https://www.inegi.org.mx/temas/empleo/).

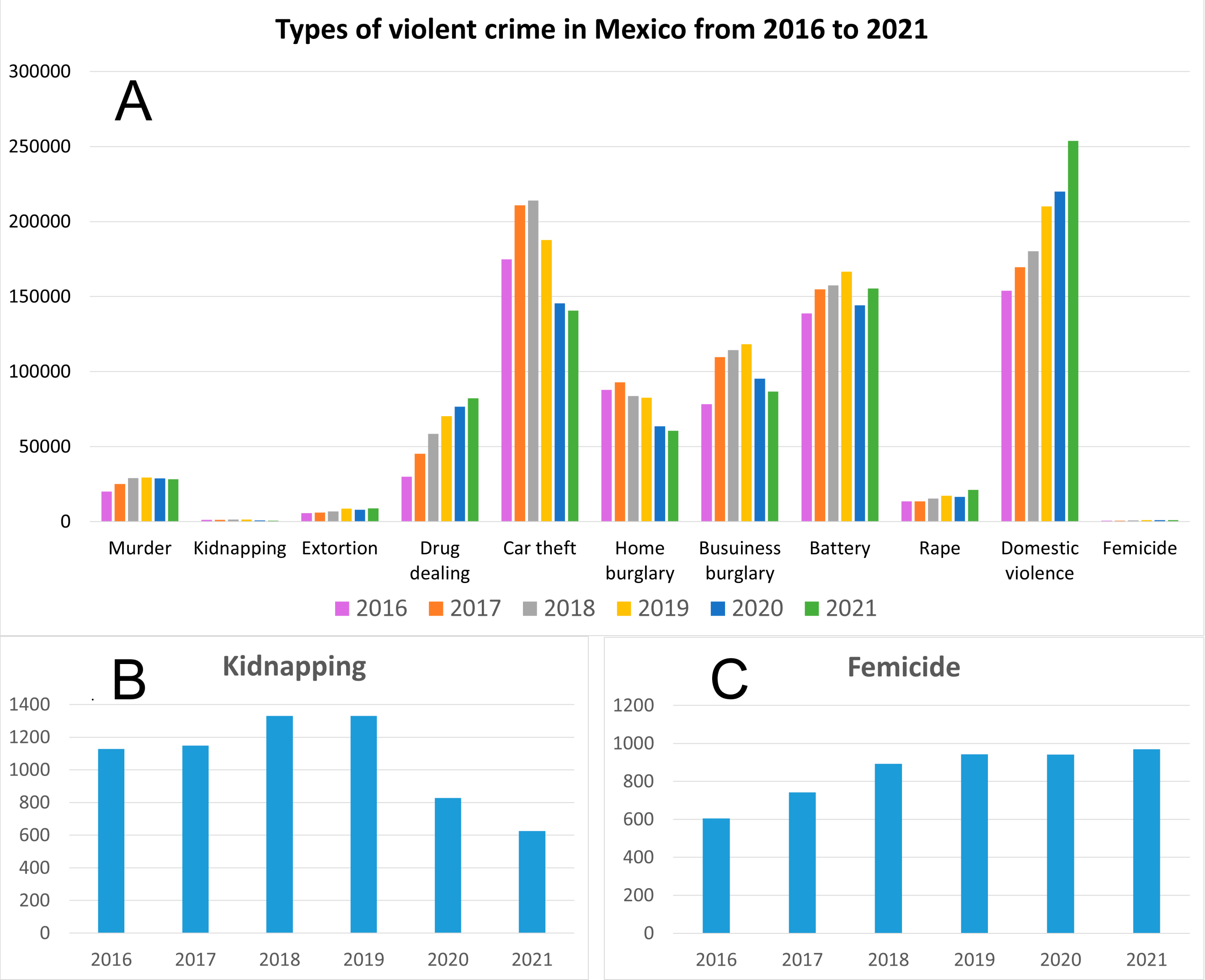

Figure S3. Total number of violent crimes per year in Mexico from 2016 to 2021. The data used to create the correlation table in Fig.2 of the main text were broken down by Federal State. Here we show (panel **A**) the total number over the Country for each of the 11 violent crimes considered. Panels **B** and **C** show kidnapping and femicide separately, as these data are not visible in A due to the large numbers of other types of crime. The data were obtained from Semáforo Delictivo.

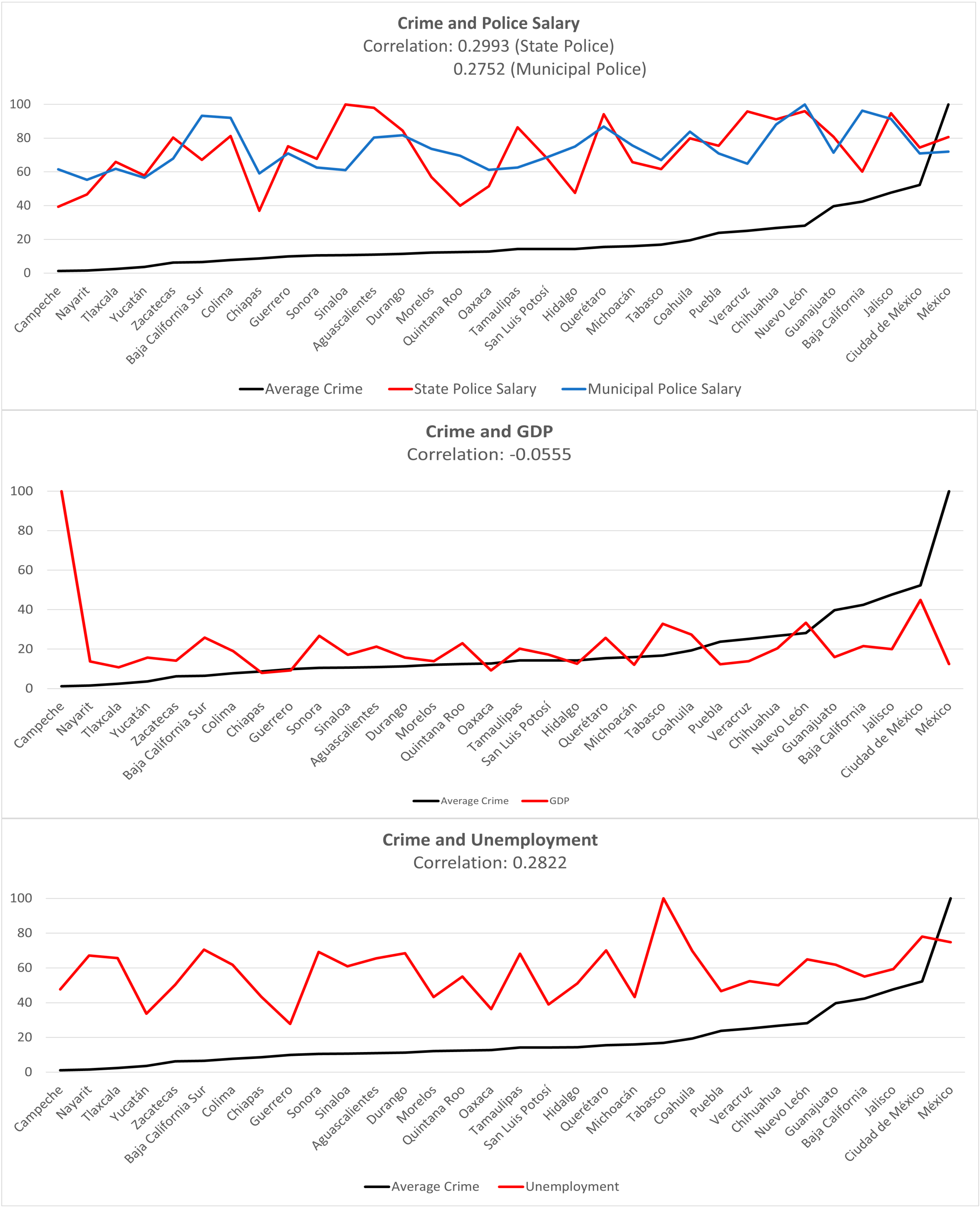

Figure S4. **Example of the data used to compute the correlation matrix** (Fig.2 of the main text). We have computed the Pearson correlation coefficient between the level of crime and different welfare indicators using data reported in the 32 Mexican states. Here we plot representative examples for the cases of police salary, Gross Domestic Product, and unemployment. For comparison purposes, all the data have been rescaled from 0 to 100. The correlation coefficient is shown in each plot.

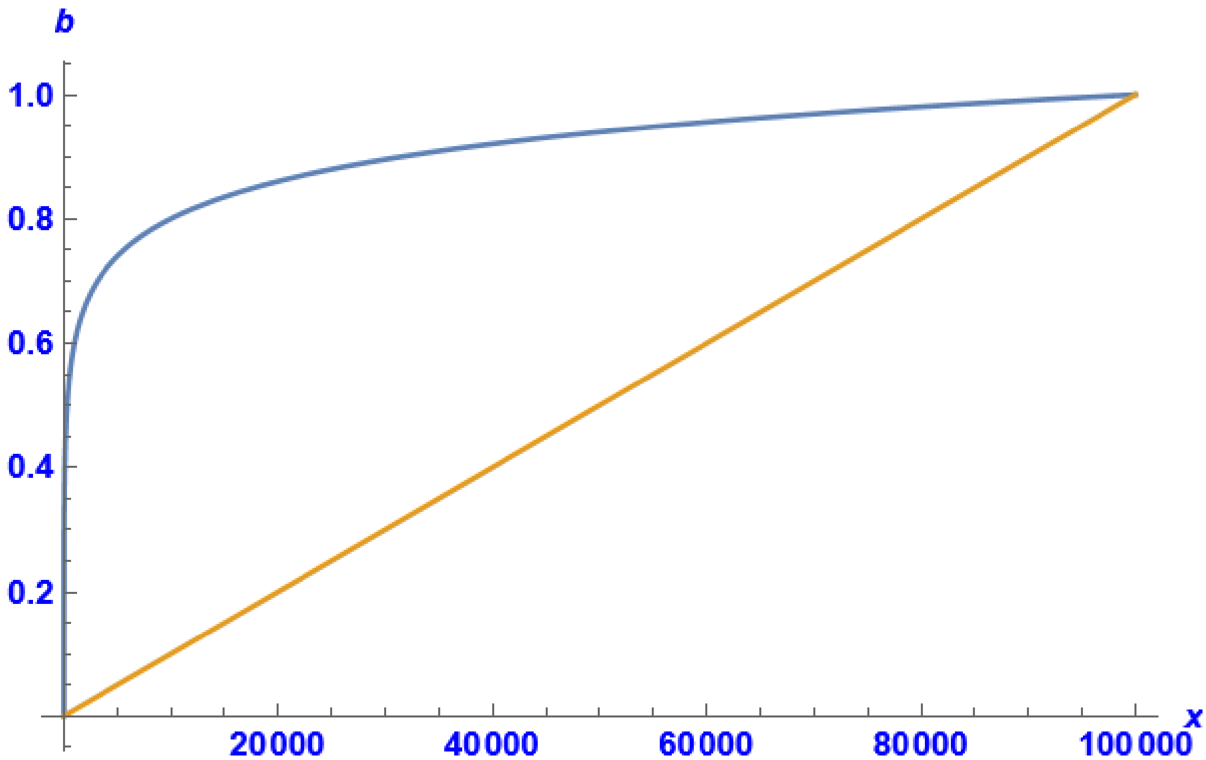

Figure S5. Logarithmic bribe. The parameter b defined in Eq. (2) of the main text represents the capacity of the criminal to bribe police officers. Since in our model the police officers' corruption thresholds are normalized in the interval $[0,1]$, the bribes b must also be normalized within this interval. Here we plot the bribe $b(x)$, defined in Eq.(2) of the main text, as a function of the criminal's money x (blue line). Using a logarithmic function allows the criminal to accumulate money faster than using a linear function (orange line). In this way, the criminal rapidly increases his ability to bribe the police, staying longer in the society. However, after a while the logarithmic function curves down, which means that after an initial period of rapid increase, the criminal's bribe capacity starts to saturate. The logarithmic function thus has two advantages: first, it makes the simulation reach a stationary state faster than a linear function, and second, after an initial period of rapid growth, it saturates, which is consistent with intuition. We do not have information regarding how fast criminals accumulate money (since this is illegal money, there is no official record about the rate at which criminals increase their fortunes).

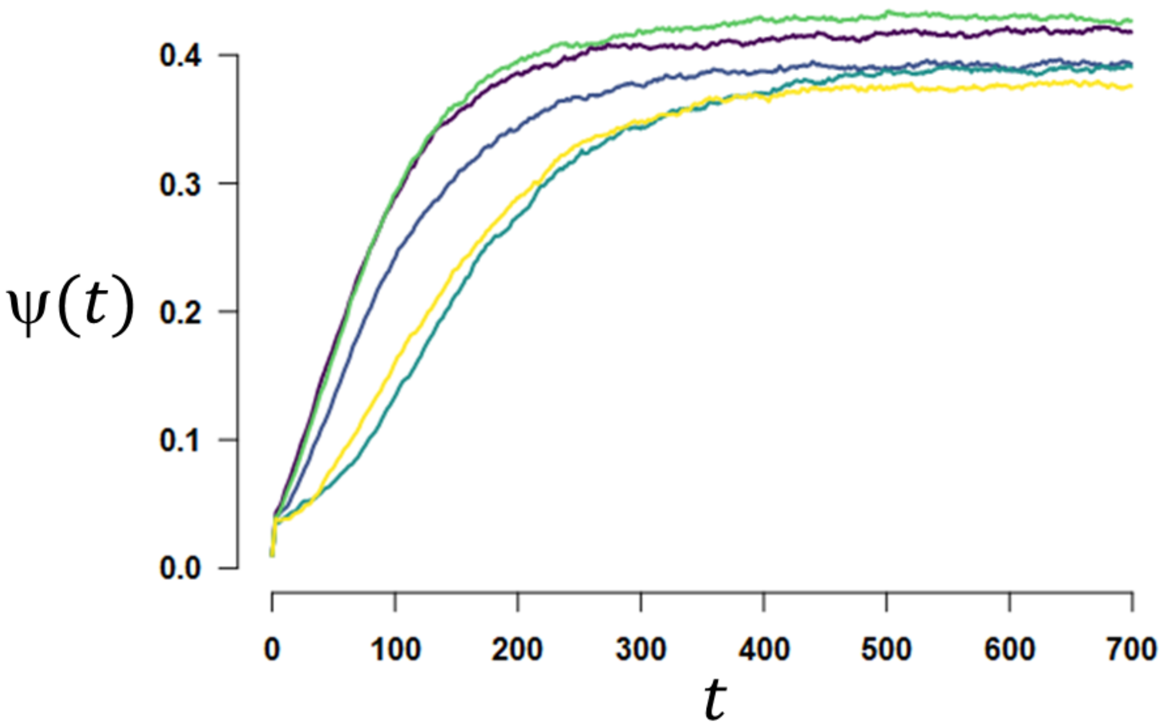

Figure S6. Order parameter as a function of time. The order parameter is defined as $\psi(t) = N_v(t)/N$, where $N_v(t)$ is the number of victims of crime at time t and N is the total number of agents in the system. Note that after a transient time (about 500 time steps) the order parameter reaches a stationary value. To smooth out the fluctuations we define the stationary value of the order parameter as in Eq.(4) of the main text: $\psi = \frac{1}{T_m - T_0} \sum_{t=T_0}^{T_m} \psi(t)$, where T_0 is the transient time and T_m the maximum computing time. In this work we set $T_0 = 500$ and $T_m = 1000$. Each curve in this figure corresponds to one realization of the dynamics. To compute ψ, in addition to averaging over time, we also averaged over 10 realizations.

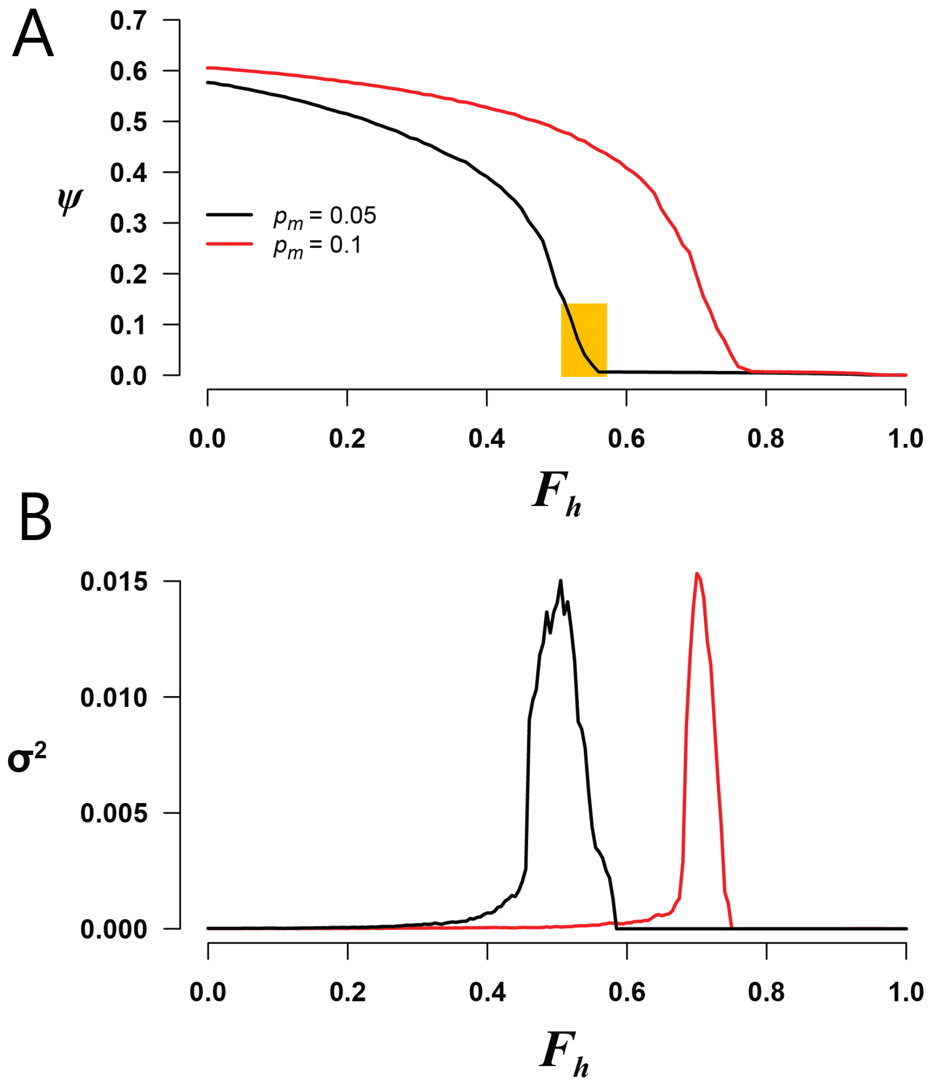

Figure S7. Variance of the order parameter and the nature of the phase transition. (A) Phase transition driven by the fraction F_h of honest police officers for two values of the turning probability p_m that a regular citizen turns into a criminal. These are the same curves as the ones displayed in Fig. 4A of the main text. The small orange rectangle illustrates the region bounded between 1.5% and 15% of criminality, which seems to correspond to the crime incidence observed in Mexico. Note that this region is very close to the critical point. **(B)** Variance of the order parameter as a function of F_h. Note that the variance peaks at the values of F_h at which the phase transition occurs. This result suggests that the phase transition driven by F_h is of second order.

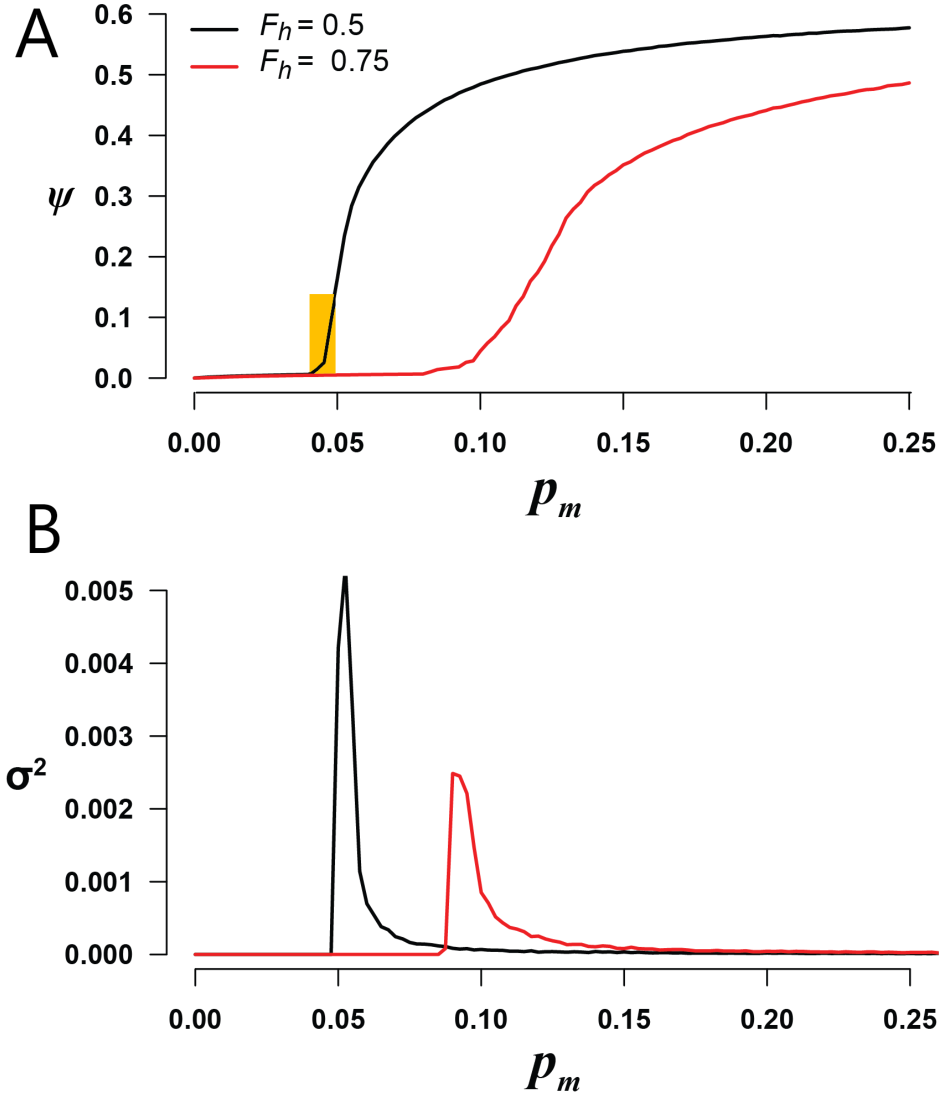

Figure S8. Variance of the order parameter and the nature of the phase transition. (A) Phase transition driven by the turning probability p_m that a regular citizen becomes a criminal, for two different values of the fraction F_h of honest police officers. These are the same curves as the ones displayed in Fig. 4B of the main text. The small orange rectangle illustrates the region bounded between 1.5% and 15% of criminality, which seems to correspond to the crime incidence observed in Mexico. Note that this region is very close to the critical point. **(B)** Variance of the order parameter as a function of p_m. Note again that the variance shows sharp peaks at the values of p_m at which the phase transition occurs. The results shown in the previous and this figure suggest that the phase transition driven by F_h and p_m is indeed of second order, although a more careful analysis has to be done to precisely determine the nature of the phase transition.

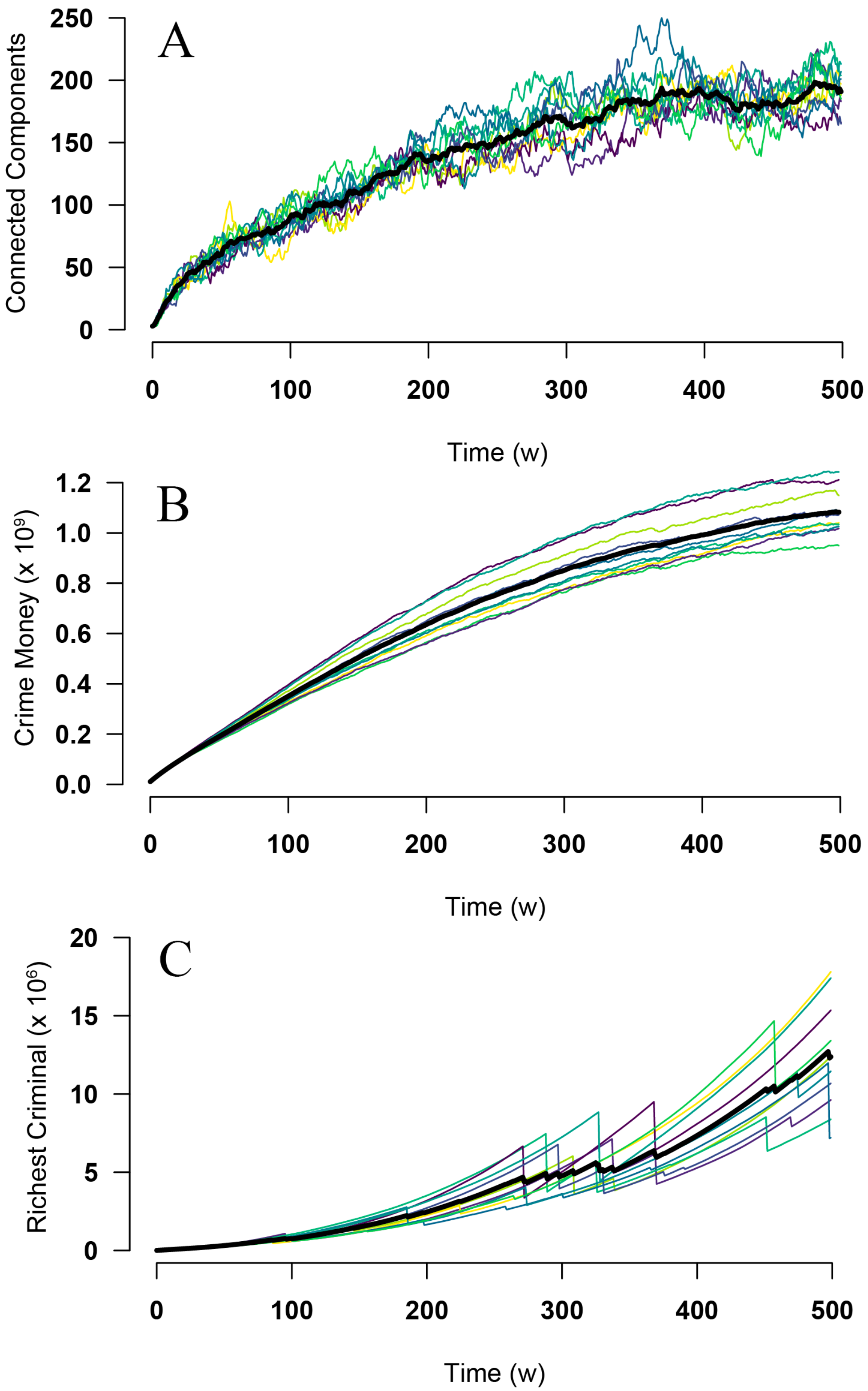

Figure S9. Evolution of the money of the criminal network. **(A)** Number of independent subnetworks (cells) of the criminal network as police officers capture criminals (same as Fig. 5E of the main text). **(B)** Despite the fracturing of the criminal network, the total accumulated money keeps increasing. **(C)** The capital of the wealthiest criminal in the network also increases. The discontinuous jumps correspond to events in which the richest criminal of the network is captured. Then, the second wealthiest criminal takes over. The different curves correspond to ten distinct realizations of the dynamics with $N = 10^5$ agents. The solid black curve is the average of these realizations.
